# MicroRNA dysregulational synergistic network: discovering microRNA dysregulatory modules across subtypes in non-small cell lung cancers

**DOI:** 10.1186/s12859-018-2536-0

**Published:** 2018-12-21

**Authors:** Nhat Tran, Vinay Abhyankar, KyTai Nguyen, Jon Weidanz, Jean Gao

**Affiliations:** 1 Department of Computer Science and Engineering, The University of Texas at Arlington, Arlington, TX, 76019 USA; 2 UTARI Research Institute, The University of Texas at Arlington, 7300 Jack Newell Blvd S, Fort Worth, TX, 76118 USA; 3 Department of Bioengineering, The University of Texas at Arlington, Arlington, TX, 76019 USA; 4 Department of Biology, The University of Texas at Arlington, Arlington, TX, 76019 USA

**Keywords:** microRNA dysregulation, Differential analysis, Biomarker discovery, Scale-free network, Synergistic module

## Abstract

**Background:**

The majority of cancer-related deaths are due to lung cancer, and there is a need for reliable diagnostic biomarkers to predict stages in non-small cell lung cancer cases. Recently, microRNAs were found to have potential as both biomarkers and therapeutic targets for lung cancer. However, some of the microRNA’s functions are unknown, and their roles in cancer stage progression have been mostly undiscovered in this clinically and genetically heterogeneous disease. As evidence suggests that microRNA dysregulations are implicated in many diseases, it is essential to consider the changes in microRNA-target regulation across different lung cancer subtypes.

**Results:**

We proposed a pipeline to identify microRNA synergistic modules with similar dysregulation patterns across multiple subtypes by constructing the MicroRNA Dysregulational Synergistic Network. From the network, we extracted microRNA modules and incorporated them as prior knowledge to the Sparse Group Lasso classifier. This leads to a more relevant selection of microRNA biomarkers, thereby improving the cancer stage classification accuracy. We applied our method to the TCGA Lung Adenocarcinoma and the Lung Squamous Cell Carcinoma datasets. In cross-validation tests, the area under ROC curve rate for the cancer stages prediction has increased considerably when incorporating the learned microRNA dysregulation modules. The extracted modules from multiple independent subtypes differential analyses were found to have high agreement with microRNA family annotations, and they can also be used to identify mutual biomarkers between different subtypes. Among the top-ranked candidate microRNAs selected by the model, 87% were reported to be related to Lung Adenocarcinoma. The overall result demonstrates that clustering microRNAs from the dysregulation pattern between microRNAs and their targets leads to biomarkers with high precision and recall rate to known differentially expressed disease-associated microRNAs.

**Conclusions:**

The results indicated that our method improves microRNA biomarker selection by detecting similar microRNA dysregulational synergistic patterns across the multiple subtypes. Since microRNA-target dysregulations are implicated in many cancers, we believe this tool can have broad applications for discovery of novel microRNA biomarkers in heterogeneous cancer diseases.

## Background

Lung cancer accounts for more than 1.5 million deaths globally per year and is the leading cause of cancer-related mortality. About 87% of the lung cancer cases are classified as Non-Small Cell Lung Cancer, and the 5-year survival rate of all stages is below 17% because the majority of lung cancer patients (57%) are diagnosed at later stages because early disease is typically asymptomatic [1]. Even when diagnosed early, the only recommended treatment is surgical resection, despite that up to 30% of those successfully treated will still die within five years of initial diagnosis [2]. Therefore, the development of early diagnosis and treatment strategy is critical and essential for the control of this deadly disease. Recently, it has been found that microRNAs have the potential as both biomarkers and therapeutic targets for lung cancer [3, 4].

MicroRNAs (miRNAs) are a recently discovered class of small noncoding RNA. Approximately 22nt, miRNAs post-transcriptionally target messenger-RNAs (mRNAs) to regulate the translation of target genes. They have been found to play a critical role in various biological functions such as proliferation, differentiation, and apoptosis [5]. Thus, abnormal miRNA regulatory events can cause a significant impact on various cellular functions, ultimately resulting in complex events leading to cancer. Increasing evidence suggests that miRNAs can have a causal role in tumorigenesis [6].

Due to the significant role of miRNAs found in cancer biology, many existing lung cancer studies use miRNA expression profiles for accurate prediction of lung cancer stages or subtypes [7, 8]. In a typical differential expression analysis, a univariate statistical method (e.g., student’s *t*-test, false discovery rate threshold) is performed to select miRNAs with a significant deviation between normal and tumor sample groups. However, the results are not always satisfactory, as large-scale multi-omics analysis of non-small cell lung adenocarcinoma (LUAD) revealed distinct interactions of miRNA to target mRNA that are specific to histological subtypes [9]. In other words, an identified miRNA biomarker may correctly classify tumor based on analyses done on one particular subtype but may misclassify cases of other subtypes, where it may target a different set of mRNAs. Therefore, for a more robust selection of miRNA biomarker, analysis of the deviation in miRNA-target interactions between various lung cancer subtypes should be considered to assess their potential as predictor to this heterogeneous disease.

Experimental evidence has shown that multiple miRNAs can potentially target a gene through synergism, in which two or more miRNAs can cooperatively co-regulate an individual gene [10]. Studying the synergism of miRNAs within a specific cellular environment is another critical step to determine their disease-specific functions at the system level. Construction of the miRNA co-regulation network by considering regulatory targets with similar functions [11] revealed a miRNA-miRNA functional synergistic network; however, the study of the changes in miRNA-target interactions between different cancer subtypes has mainly left uncovered.

To further our understanding of the role of miRNAs in lung cancers, we aim to identify differentially expressed miRNAs while considering miRNA-target dysregulations among different cancer subtypes. We extended the brilliant miRNA-target dysregulation idea from Xu et al. [12] and proposed a novel miRNA clustering strategy to identify miRNA dysregulatory modules. We hypothesize that by identifying the context-specific group structures among the miRNAs, the differential analysis procedure can benefit from a more robust selection of miRNA biomarkers that can accurately predict cancer stages across different subtypes.

## Methods

### Dataset and notations

We denote the miRNA and mRNA expression profiles as column vectors $\mathbf {x_{i}}=\left [x_{i}^{1}, x_{i}^{2},\ldots,x_{i}^{s}\right ]^{\top }$ and $\mathbf {y_{j}}=\left [y_{j}^{1}, y_{j}^{2},\ldots,y_{j}^{s}\right ]^{\top }$ to represent the expression level of miRNA *i* and mRNA *j* across *s* samples, respectively. To represent miRNA and mRNA expressions for a specific group of samples, we denote column vectors $\mathbf {x_{i}^{C}}=\left [x_{i}^{1}, x_{i}^{2}, \ldots, x_{i}^{n_{C}}\right ]^{\top }$ and $\mathbf {y_{j}^{C}}=\left [y_{j}^{1}, y_{j}^{2}, \ldots, y_{j}^{n_{C}}\right ]^{\top }$, respectively, where *n*_*C*_ is the number of samples attributed with a particular phenotype *C*, e.g., normal, stage I cancer, stage II cancer, etc. Note, boldface variables are to represent vectors and non-boldface for scalars. Also, for expression data, we use subscripts to identify a specific miRNA or mRNA expression level, and superscripts to identify a sample group.

### Identification of miRNA biomarkers for lung cancer

**Fig. 1:**
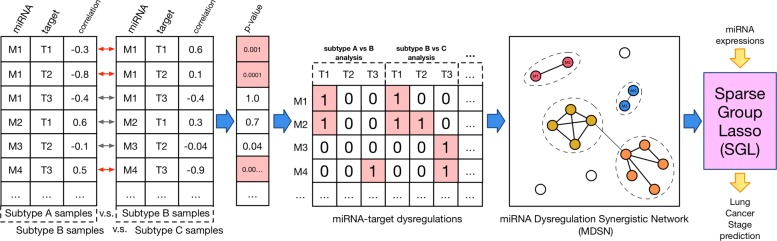
Overview of the MiRNA Dysregulational Synergism Network pipeline. In the first step, multiple differential analyses between different subtype groups identified miRNA-target dysregulations. Then, the miRNA dysregulations across multiple subtype analyses is used to form the miRNA-target dysregulation association matrix. Next, the MDSN network is constructed by computing miRNA-miRNA similarity measures, and is used to extract miRNA modules by a graph partitioning method. Finally, provided the extracted miRNA modules, the Sparse Group Lasso performs classification of the cancer stage given a sample’s miRNA expression profile

As an overview of our pipeline, illustrated in Fig. 1, we developed a novel approach to identify miRNA dysregulation modules by detecting changes in miRNA-target associations between different cancer subtypes. First, we identify significant deviations in miRNA-target correlations between two sample groups. For each miRNA-target pair found significantly deviated, we form a connection to build a miRNA-target dysregulation association matrix. From the identified miRNA-target dysregulations, miRNA modules are extracted such that functionally similar miRNAs belong in the same module if they dysregulate similar targets across multiple cancer subtypes. To accomplish this, a miRNA-miRNA Dysregulational Synergism Network (MDSN) is constructed, and a graph partitioning method is applied to identify significant miRNA modules. At the final step, classification analysis predicts cancer stage and selects relevant biomarkers only from miRNA expression profile data. A Sparse Group Lasso regularization is applied with the intuition that if a miRNA is relevant, the rest of miRNAs in the same module are probably also relevant.

### Step 1: Identifying miRNA-target dysregulations between subtypes

For every putative miRNA-target pairs, we incorporated sample-matched miRNA expression and mRNA expression data from distinct sample groups to identify aberrant miRNA-target interactions. More specifically, the aim is to find regulatory changes by differential analysis of the miRNA-target pair’s correlation values between two sample groups of different lung cancer subtypes. This Dysregulation criterion was proposed by Xu et al. [12], which defines the difference of the Pearson’s correlations between a tumor and a non-tumor group for miRNA *i* and target *j* as:

1 $$ {Dys}_{ij}^{AB} = \frac{\text{cov}\left(\mathbf{x_{i}^{A}},\mathbf{y_{j}^{A}}\right)} {(n_{A}-1)\sigma_{x_{i}^{A}}\sigma_{y_{j}^{A}}} - \frac{\text{cov}\left(\mathbf{x_{i}^{B}},\mathbf{y_{j}^{B}}\right)} {(n_{B}-1)\sigma_{x_{i}^{B}}\sigma_{y_{j}^{B}}} \\  $$

where $\sigma _{x_{i}^{A}}$ and $\sigma _{x_{i}^{B}}$ denote the standard deviation of miRNA *i* expressions of sample groups *A* and *B*, respectively. To determine whether the deviation of the correlation between the two groups is significant, Xu et al. randomly assigned patients to the two groups and recalculated *Dys* 10,000 times, and obtained a *p*-value by the frequency of the random *Dys* being higher than the actual *Dys*.

To improve the computational performance of obtaining a significance value for the deviation between two correlation coefficients, we instead applied Fisher’s transformation [13] as utilized in our previous publication [14]. To summarize, for a given miRNA *i* and target *j*, we calculated the two Pearson’s correlation values *r*_*A*_ and *r*_*B*_ from each sample group then obtained their corresponding z-values *z*_*A*_ and *z*_*B*_ through Fisher’s transformation $z=\frac {1}{2}\ln \left (\frac {1+r}{1-r}\right)$. The z-value for the difference between *z*_*A*_ and *z*_*B*_ is obtained by

$$z_{AB}=\frac{z_{A}-z_{B}}{\sqrt{1/(n_{A}-3)+1/(n_{B}-3)}} $$

Finally, we can convert the absolute value of *z*_*AB*_ to a *p*-value (two-tailed) and thereby obtain a statistical significance of the difference between two miRNA-target correlations. The cut-off for the *p*-value threshold was chosen at 0.001, as it has been commonly used as a threshold in several correlation studies.

### Step 2: Building the miRNA-target dysregulation association matrix

One primary function of miRNAs is the cleavage of the transcript of its target gene to regulate gene expression. Thus, in the task of identifying aberrant miRNA-target interactions, the inverse correlation should be a prerequisite for candidate miRNA and target pairs to avoid false-positives. In other words, only miRNA-target pairs which have a negative Pearson’s correlation in at least one of the sample groups, *A* or *B*, were considered.

Furthermore, since the primary goal of this study is to discover novel miRNA biomarkers to help understand cancer stage progression, it is essential to consider as many miRNAs as possible. In this study, the miRNA-target relationship prediction algorithms, e.g., TargetScan 7.1 [15] and miRanda [16], were not utilized as the interaction databases only covered a total of 263 miRNAs out of 1881 miRNAs present in the miRNA expression profiles.

For each putative miRNA *i* and target *j* considered, we repeated the dysregulation analysis procedure in Step 1 between all pairs of different lung cancer subtypes as independent dysregulation analyses. Then, all miRNA-target dysregulations found significant were encoded by constructing a matrix **A** with entry *A*_*ij*_ equal to 1 if the *p*-value of the miRNA *i* and target *j* dysregulation passes the *p*-value threshold and 0 otherwise. For each independent dysregulation analyses, the matrix **A** is concatenated. This matrix is interpreted as a new feature set, where each row characterizes a miRNA’s dysregulation targets that were present across multiple cancer subtypes dysregulation analyses.

### Step 3: Calculating miRNA-miRNA dysregulation functional similarity

As it has been reported, miRNAs that are functionally similar tend to have the same targets. Using the identified miRNA-target dysregulations, we inferred the context-specific functional similarity between two miRNAs by considering their mutual dysregulated targets. The functional similarity score between two miRNAs *p* and *q* is calculated by cosine similarity, defined as

2 $$ s(p,q) = \frac{\mathbf{A}_{p.} \mathbf{A}_{q.}^{\top}}{\left\lVert{\mathbf{A}_{p.}}\right\lVert_{2} \left\lVert{\mathbf{A}_{q.}}\right\lVert_{2}}  $$

where **A**_*i*._ is a row vector indicating the dysregulated targets of miRNA *i*. The cosine similarity value ranges [0,1] and can be interpreted as the number of mutual dysregulation targets shared between two miRNAs normalized by their total connections. By calculating the similarity between every miRNA-miRNA pairs, an adjacency matrix is produced to construct a miRNA-miRNA similarity network. Since it is difficult to uncover cluster structures when the network is dense, it is necessary to prune the weaker miRNA-miRNA connections.

### Step 4: Constructing the MDSN and pruning with scale-free thresholding

The scale-free topology property exists in most biological graphs, including miRNAs [17], which indicates that the miRNA-miRNA network connections follow a power-law distribution in which more miRNAs tend to have fewer neighbors and fewer miRNAs tend to have more neighbors. A well-known framework, Weighted Gene Co-expression Network Analysis (WGCNA) is utilized to prune lower weight edges with a threshold chosen such that the graph’s scale-free property still holds while preserving as many edges as possible.

After all miRNA-miRNA pairs’ cosine similarity scores are computed, they are used as edge weights in the MDSN. This is constructed by an adjacency matrix **M** with entries *M*_*pq*_=*s*(*p,q*) for all miRNAs *p, g*. Similar to the approach used in most biological networks, the miRNA node degrees is expected to exhibit a scale-free distribution under some thresholding. We applied the hard-thresholding technique in WGCNA [18] by removing from the network any edge with weight lower than the threshold, which was chosen to be the least stringent threshold such that the degree distribution maintains a desirable power-law fitting score.

### Step 5: Identifying miRNA dysregulation modules with community detection

After pruning of the MDSN, we utilized the graph partitioning approach to extract miRNA modules by assigning miRNA nodes into communities using a modularity objective proposed in the Louvain method [19]. Using a fast greedy iterative procedure, the Louvain method assigns nodes into communities by optimization of the modularity objective, which measures the density of links inside communities compared to links between communities.

To summarize the algorithm, initially, each node is assigned to its own community. At the first phase, node *i* consider each of its neighbor *j* and evaluate the gain of modularity if *i* is placed in *j*’s community, and then selects the neighbor *j* with the maximum modality gain. This first phase repeats iteratively until convergence. The algorithm then alternates to the second phase to build a new network whose nodes are the newly formed communities found in the first phase. The first and second phase are repeated iteratively until there is only one community that includes all nodes. In the final result, the algorithm gives a hierarchical community structure of all nodes in the MDSN network. The partition in this dendrogram with the highest modularity value by the Louvain algorithm is selected as the miRNA modules assignment.

### Step 6: Classification of cancer stage with identified miRNA modules

It is known that a classifier with *ℓ*- 1 norm regularization is typically used for feature selection in problems with "small n, large p." However, for problems known to have grouped features, adding group information as prior knowledge can improve feature selection and classification performance. We applied a multi-class logistic classifier with Sparse Group Lasso (SGL) with the intuition that if a miRNA predictor to cancer stage is found relevant, other miRNAs in the same group are also likely relevant since they share similar dysregulation targets across the cancer subtypes.

SGL is a linear logistic classifier with combined *ℓ*- 1 and Group Lasso *ℓ*- 2 norm regularization to achieve a sparse solution at both the group and within group level [20]. We used an indicator vector *c*_*i*_∈{0,1}^*k*^ to represent the *i*^*th*^ sample’s reported cancer stage. In this study, *k* is 5, indicating whether a sample is labeled as normal, stage I, II, III, or IV. The objective function is as follows:

3 $$ \min_{W} \frac{1}{s} \sum_{i=1}^{s} \log\left(\!1 + e^{-c_{i}(W^{\top}\mathbf{x^{i}})}\right) + \lambda \alpha ||W||_{1} + \lambda (1-\alpha) GL(W)  $$

where *λ* is the sparsity coefficient, *α* is the mixing coefficient between *ℓ*- 1 and Group Lasso *ℓ*- 2 norm, which is defined as:

4 $$ GL(W)=\sum_{g=1}^{G} \sqrt{|g|} \cdot ||W_{g}||_{2}  $$

where |*g*| is the size of the group. The Python package *pylearn*-*parsimony* was used to train the logistic regression classifier with SGL regularization.

## Result

### Applications in TCGA non-small cell lung adenocarcinoma dataset

We downloaded miRNA and mRNA expression data of the LUAD cohort from The Cancer Genome Atlas (TCGA) [9], utilizing the TCGA-Assembler tool [21]. Expression quantitation of miRNAs was calculated from the BCGSC miRNA profiling pipeline. The mRNA expression profiles were obtained using Illumina HiSeq RNA-Seq (v2). The Read Per Million miRNA Mapped (RPKM) values were log2 transformed and scaled to zero-mean and standard deviation. In total, there were 1881 miRNA expressions and 20,484 mRNA expressions profiled. The sample size characteristics of LUAD subjects are shown in Table 1.

**Table 1:** Sample size characteristics of the TGCA LUAD dataset

Phenotype	Sample size
Normal (matched)	20
Stage I	277
Stage II	121
Stage III	84
Stage IV	24
Acinar^a^	18
Bronchioloalveolar^a^	24
Clear Cell	2
Colloid^a^	10
Micropapillary	3
Mucinous	2
Papillary^a^	23
Signet Ring	1
Solid	5
Mixed subtype	107
Not otherwise specified	320

^a^Histological subtypes selected for dysregulation analysis for their sufficient sample size

#### Identified miRNA-target dysregulations between LUAD subtypes

We identified significant dysregulations for every miRNA-target pair between 1881 miRNAs and 20,484 mRNAs. Each miRNA-target pair is tested for significant change in correlations between different subtype sample groups. Due to insufficient sample size in some subtypes, only four histological LUAD subtypes were selected for subtypes dysregulation analysis, as outlined in Table 1. To build the miRNA-target dysregulation matrix, we performed an independent dysregulation analysis for each pair-wise combination of the four subtypes.

Setting the *p*-value threshold parameter at *p*<0.001, we obtained a sum of 1,896,631 miRNA-target dysregulations from a union of six independent dysregulation analyses for the Acinar, Bronchioloalveolar, Colloid, and Papillary subtypes. In other words, we identified miRNA-target dysregulations between Acinar vs. Bronchioloalveolar, Bronchioloalveolar vs. Colloid, Acinar vs. Colloid, and so on. Since it is very likely that false-positives exist among the identified miRNA-target dysregulations, we accounted for this by careful selection of the threshold parameter to prune weaker miRNA synergism similarities.

#### Selection of threshold parameter for the scale-free topology of MDSN for LUAD cohort

After identifying miRNA-target dysregulations among the lung cancer subtypes, we computed the miRNA-miRNA cosine similarity score for every pair of miRNAs to construct the MDSN. For every pair of the 1314 miRNAs (found dysregulated), we computed a total of 754,086 cosine similarity scores. The power law fitting score [18] is defined as *corr*(*log*_10_(*s*),*log*_10_(*p*(*s*)))^2^ where *s* is the similarity scores and the distribution *p*(*s*) is modeled by a histogram of binned data samples. The *R*^2^ score computed over all miRNA-miRNA pairs was 0.9135, which satisfies the *R*^2^>0.8 criterion and indicates the network has a scale-free topology. The similarity score power parameter was kept at *β*=1.

**Fig. 2:**
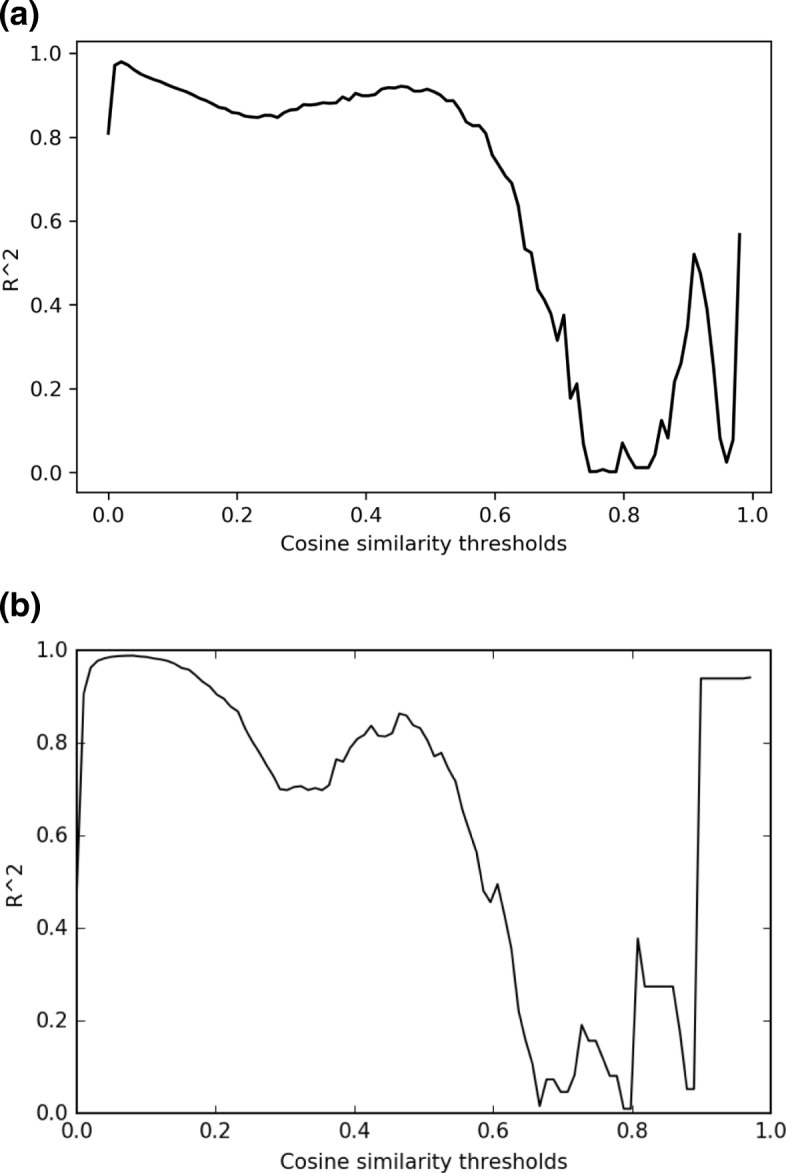
The R^2^ scale-free criterion fit score at different hard-thresholds. Edges in the MDSN are pruned if their cosine similarity score is lower than the threshold. **a** R^2^ scores under hard-thresholding for LUAD cohort. **b** R^2^ scores under hard-thresholding for LUSC cohort

**Fig. 3:**
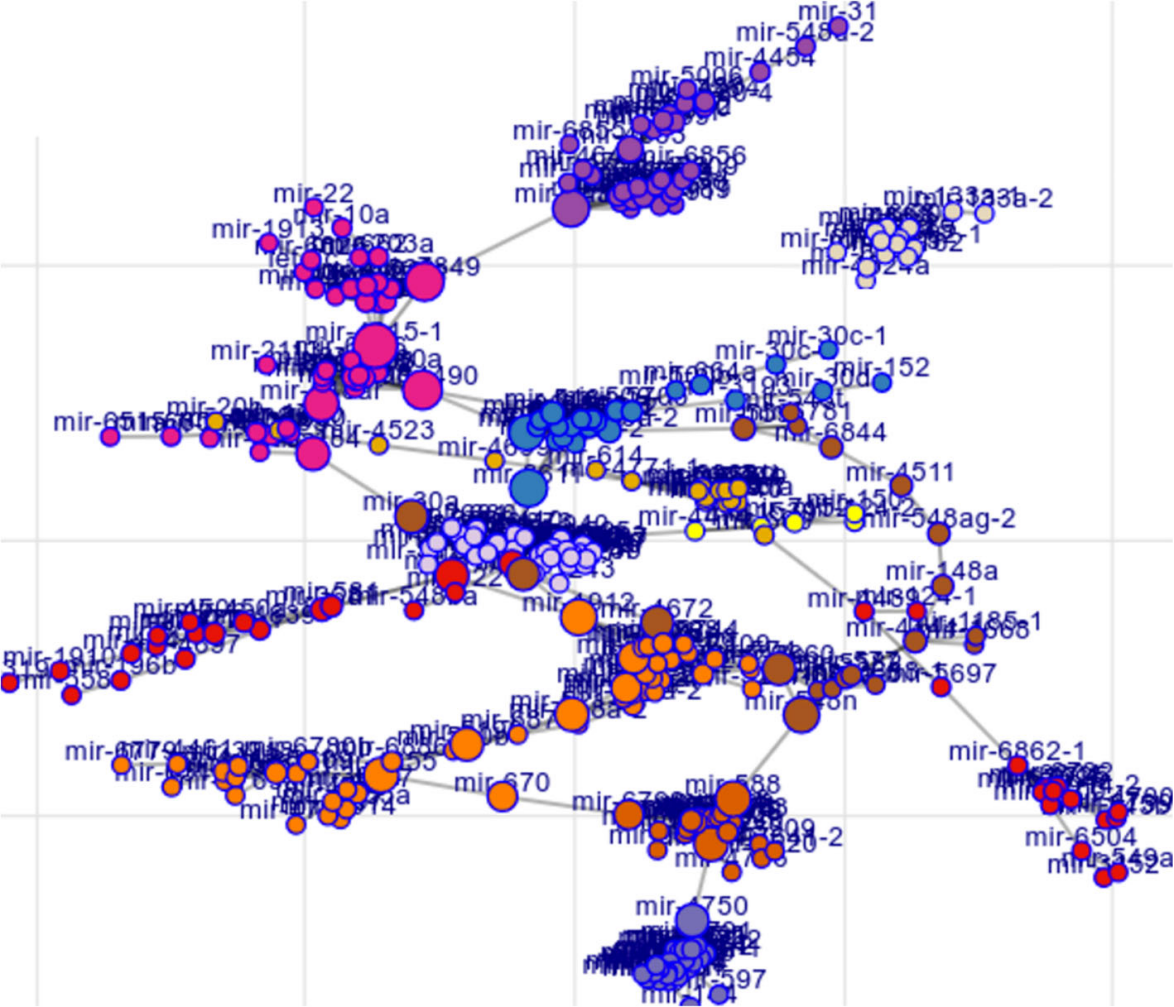
Graph force-layout of the MDSN. Nodes are positioned closer together if their interconnectivity is high. MiRNA modules assignment, denoted by node color, is determined from the Louvain community detection method which maximizes the modularity objective. It is observed that miRNAs in the same family tend to be grouped as a clique

Next, we proceeded to select a hard-threshold parameter to prune edges from the MDSN with a trade-off between maximizing the scale-free topology fit score and maintaining information in the network for modules discovery. The trade-off can be visualized in Fig. 2a. We selected the threshold at 0.55, where the scale-free topology score is above 0.8, and pruned all edges which have cosine similarity score lower than 0.55. After edge pruning, the number of non-isolate miRNA nodes remaining in the MDSN was 423. From the reduced MDSN network, we applied the Louvain community detection method to identify miRNA modules, and the assignment of miRNAs to the module is indicated by color as shown in Fig. 3.

### Applications in the TCGA lung squamous cell carcinoma dataset

We also obtained matched miRNA and mRNA expression profiles from the TCGA Lung Squamous Cell Carcinoma (LUSC) cohort [22]. The preprocessing procedure of miRNA and mRNA expression profiles are the same as in the LUAD cohort. An overview of the sample sizes and clinical characteristics is summarized in Table 2. According to the clinical data compiled by TCGA-Assembler [21], only less than 20 samples had a histologic subtype labeled, and the majority of samples were labeled as Not Otherwise Specified. Thus, we could not perform the miRNA-target dysregulation analyses from the provided LUSC histological subtypes information due to the insufficient sample size of labeled data.

**Table 2:** Sample size characteristics of the TGCA LUSC dataset

Phenotype	Sample size
Normal (matched)	37
Stage I	155
Stage II	125
Stage III	50
Stage IV	3
Lung Basaloid SCC	10
Lung Papillary SCC	5
Lung small cell SCC	2
Not otherwise specified	353
Primitive^a^	59
Classical^a^	96
Basal^a^	156
Secretory^a^	53

^a^Predicted lung squamous cell carcinoma subtypes selected for dysregulation analyses

One reason for this issue is that it has been known the lung squamous cell carcinoma is clinically and genetically heterogeneous, and it is challenging to sub-stratify this heterogeneity. However, a study by Wilkerson et al. [23] discovered reproducible and clinically significant LUSC subtypes that can be predicted from the mRNA expression profiles. A representative expression profile for each of the four subtypes, Primitive, Classical, Basal, and Secretory, were summarized by a cluster centroid consisting of 196 genes. Using the cluster centroids representing the four LUSC subtypes, we performed subtype prediction for all LUSC samples using the nearest-centroid classification algorithm proposed in [24].

#### Identified miRNA-target dysregulations between LUSC subtypes

After the subtype prediction of the LUSC samples were obtained, we tested for significant dysregulation for every miRNA-target pair between 1870 miRNAs and 20,472 mRNAs. Six independent dysregulation analyses were performed for every pairwise combination of the four subtypes, e.g., Primitive vs. Classical, Basal vs. Secretory, Primitive vs. Basal, and so on. A union of the six analyses revealed a sum of 1,560,419 miRNA-target dysregulations found at the *p*-value cut-off of 0.001.

#### Selection of threshold parameter for the scale-free topology of the MDSN for LUSC cohort

For every pair of the 1490 miRNAs found with dysregulation patterns across multiple LUSC subtypes, we computed a total of 754,086 cosine similarity scores. Similar to the procedure applied to the network in LUAD cohort, we selected the edge-prune threshold at 0.50, where the scale-free topology criterion *R*^2^ score is higher than 0.8, shown in Fig. 2b. The number non-isolate miRNA nodes that remained in the MDSN is 391.

### Extracted miRNA modules are consistent between independent subtypes dysregulation analyses

**Fig. 4:**
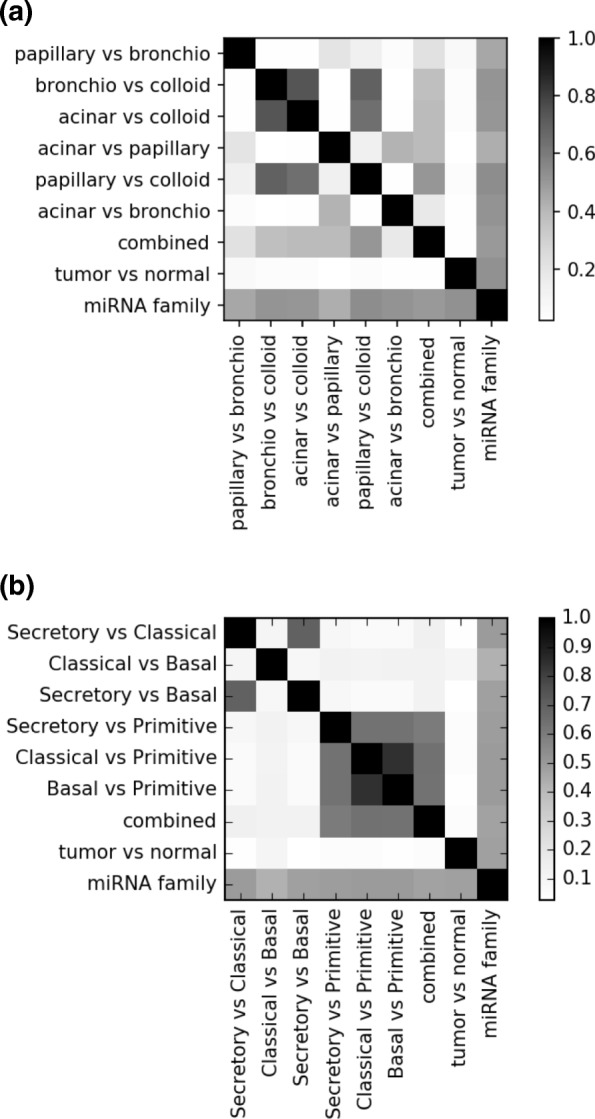
MiRNA modules similarity across independent subtypes dysregulation analyses. Each row/column in the figure indicates a miRNA module assignment, and the color squares indicate the NMI score for the agreement between two miRNA clustering assignments. For example, the “Bronchio vs Colloid” item indicates the miRNA modules assignment extracted from a MDSN derived from dysregulation analyses between subtypes Bronchioloalveolar v.s. Colloid. **a** Comparison of extracted miRNA modules from the LUAD cohort. **b** Comparison of extracted miRNA modules from the LUSC cohort

To evaluate the consistency of the extracted miRNA modules resulting from independent differential analyses, we compared the miRNA module assignments between different pairwise subtypes dysregulation analyses, combined analyses of all subtypes, normal-tumor dysregulation analysis, and miRNA family information. The score which measures the agreement between two clustering assignments is the Normalized Mutual Information (NMI) metric. As shown in Fig. 4, the extracted miRNA modules showed agreement in some of the independent subtypes dysregulation analyses for both LUAD and LUSC cohorts. For example, in Fig. 4a, after identifying dysregulations between "Bronchio vs. Colloid" subtypes and forming the MDSN, the extracted miRNA modules have a similar clusters structure to that of the modules extracted in "Acinar vs. Colloid." This may indicate the same groups of miRNA are dysregulated in the Acinar, Bronchioloalveolar, and Colloid subtypes. Similarly in the LUSC cohort shown in Fig. 4b, extracted miRNA modules identified from "Classical vs. Primitive" are highly similar to those from "Basal vs. Primitive," indicating the same groups of miRNA are dysregulated in these three subtypes. Notably, "tumor vs. normal" miRNA modules were not similar to any of the subtypes dysregulation analyses.

### Incorporating miRNA modules information improves prediction of LUAD lung cancer stage

We applied the logistic classifier with SGL using the extracted miRNA modules as prior information to the Sparse Group Lasso regularization. Using a one-vs-rest scheme for multi-class classification, SGL classifies between normal, stage I, stage II, stage III, and stage IV samples, with numbers of samples corresponding to the first column of Table 1. We empirically set the sparsity parameters *λ*=1.0 and *α*=0.5 that were found to give the best prediction performance from 5-fold cross-validation tests.

**Fig. 5:**
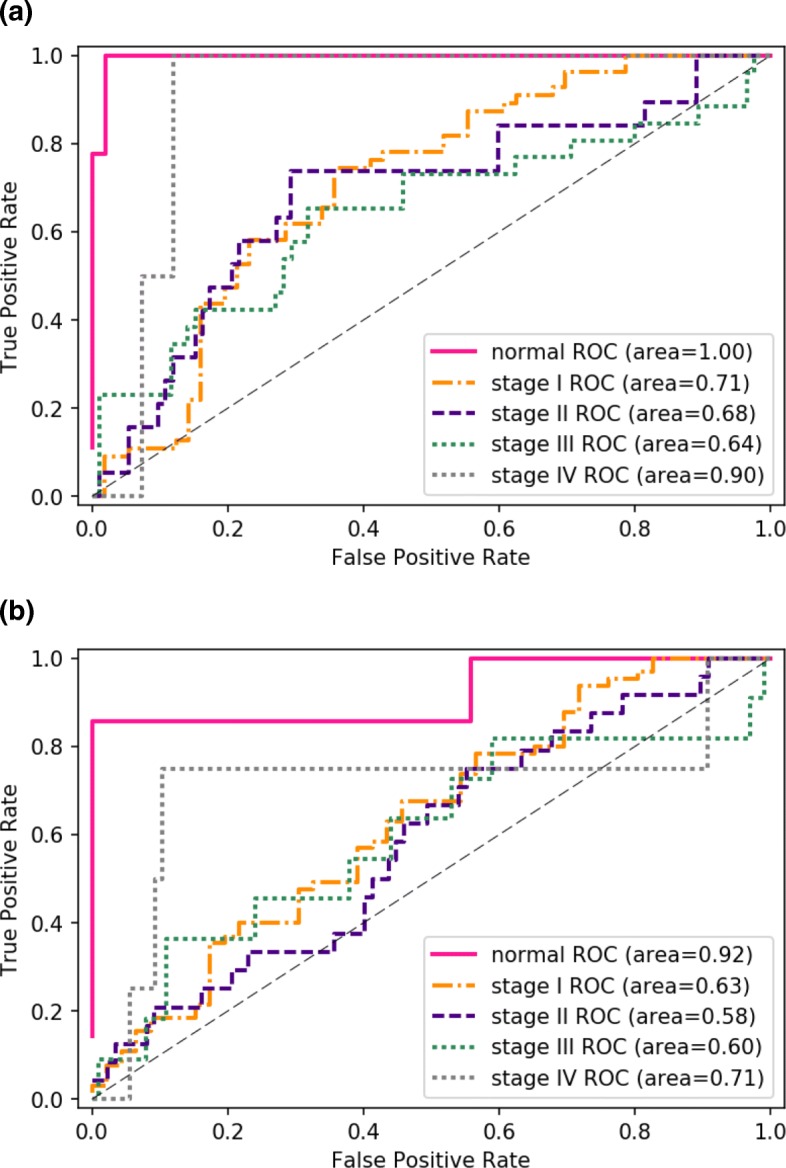
ROC area under the curve scores for prediction of LUAD stages. Comparison result in multi-stage classification performance shows improved accuracy when incorporating learned miRNA modules to the SGL classifier. **a** ROC curves for SGL with miRNA modules. **b** ROC curves for L1 regularized logistic regression

To assess whether adding miRNA clusters information improves stage prediction performance, we compared cross-validation scores between SGL and a logistic regression classifier with only *ℓ*- 1 regularization. With each classifier, we computed the area under the ROC curve rates for each stage from a train-test split of 20%, as shown in Fig. 5.

### MicroRNA groups lead to higher recall and precision of candidate miRNA biomarkers

**Fig. 6:**
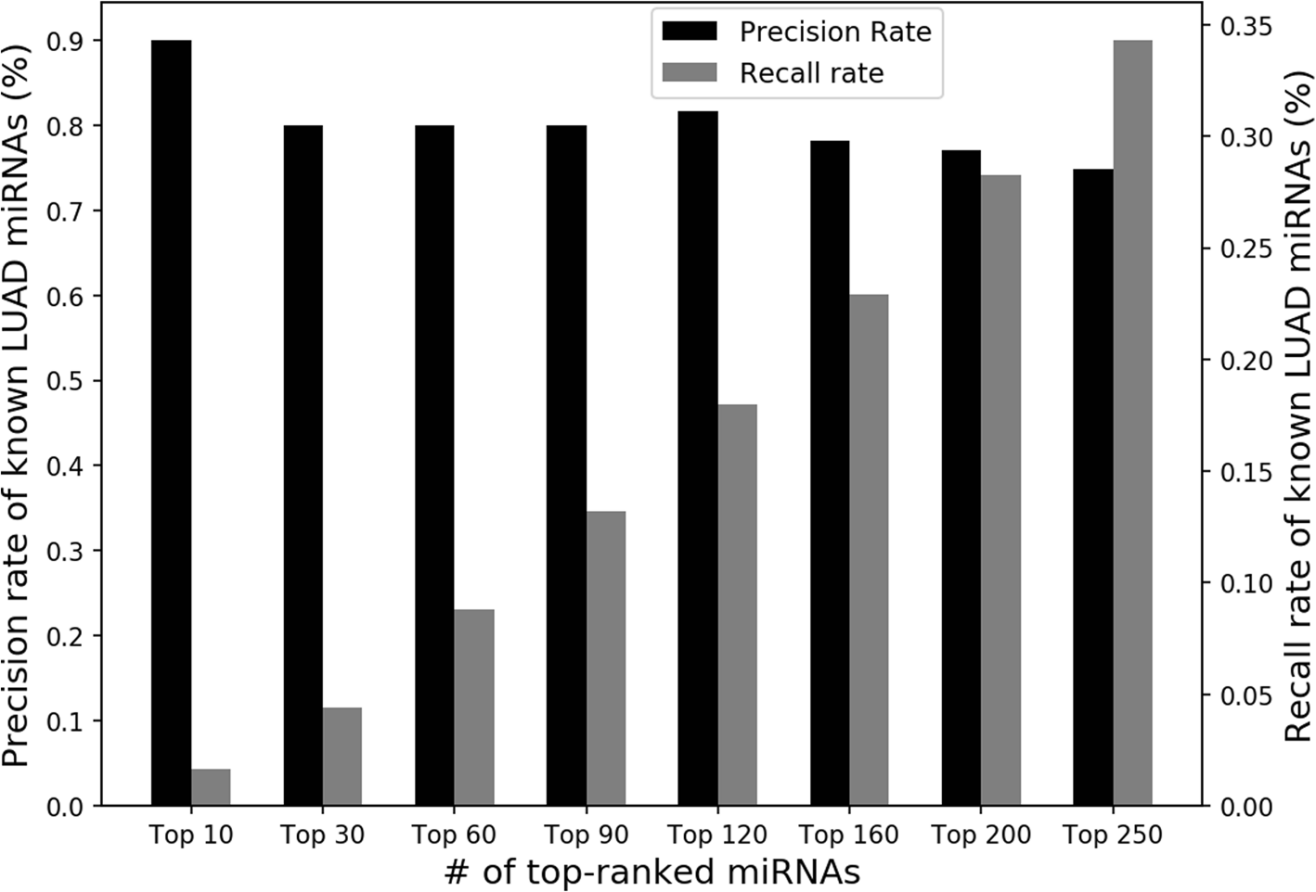
Precision and recall rates of candidate miRNAs selected by SGL. Among all 246 candidate miRNAs selected by SGL to classify normal vs. tumor, we selected *k* top-ranked miRNAs by sorting top *k* coefficients by absolute value. The left y-axis (black bars) represents the percentage of known LUAD miRNAs in the top-ranked set. The right y-axis (gray bars) represents the percentage of miRNAs recalled from known LUAD miRNAs

To validate whether the extracted miRNA modules aid the SGL classifier in selecting relevant miRNA biomarkers, we investigated how many of candidate miRNA biomarkers selected are known LUAD-associated miRNAs. We utilized a benchmark database of differentially expressed LUAD miRNAs from the dbDEMC [25]. Last updated June 2014 as of this writing, the dbDEMC contains 545 miRNAs reported by high-throughput experiments to be differentially expressed in LUAD. In a normal vs. tumor binary classification experiment using SGL which incorporates the extracted miRNA modules, we showed high precision and recall rates of top-ranked candidate miRNAs to known differentially expressed LUAD miRNAs from the dbDEMC database in Fig. 6.

## Discussion

In this study, we integrated paired miRNA and mRNA expression data to detect aberrant miRNA-target interactions between lung cancer subtypes to discover novel miRNA biomarkers to predict lung cancer stages. We have developed an efficient method to identify dysregulations among millions of potential regulatory relationships between 1,881 miRNAs and more than 20,000 mRNAs across multiple lung cancer subtypes. Among all the regulatory relationships considered, 4.9% of the miRNA-target pairs were found to have aberrant behavior across the different subtypes of the lung cancer diseases. Since the LUAD and LUSC are clinically and genetically heterogeneous diseases, utilizing this information would provide a glimpse into the miRNAs’ role in cancer pathogenesis in some specific lung cancer subtypes. This was apparent in Fig. 4, where it is apparent that some specific lung cancer subtypes possessed similar groups of dysregulated miRNA modules across multiple independent subtypes dysregulation analyses. For instance, note that the Primitive subtype in LUSC has high NMI values between the Secretory vs. Primitive, Classical vs. Primitive, and Basal vs. Primitive analyses. This indicates that in the Primitive subtype samples, there are possibly a few groups of miRNAs that have a consistent set of dysregulated targets, exclusive to all other LUSC subtypes. It would be interesting to report an analysis on such group of miRNA-target dysregulations in this Primitive subtype, which coincidently has the worst survival outcome (*p*<0.05) than the other three subtypes [23]. Such an observation may not be apparent with only a normal vs. tumor differential analysis, as it is shown in Fig. 4 where the NMI values are near zero in the normal vs. tumor dysregulation analysis compared to all other subtypes dysregulation analyses.

Despite that a growing number of miRNAs have been rigorously studied, the functions of most miRNAs are still unknown. Furthermore, only a small fraction of miRNAs were considered in the target prediction algorithms that provide a database of putative miRNA-mRNA relationships. By considering all potential miRNAs and their targets, our method can be used for novel miRNA functions discovery. However, a primary concern of this task is that selection of various thresholding hyper-parameters may produce unstable results. We performed the miRNA-target dysregulation analysis with varying *p*-value threshold at 0.01 and 0.001 and found similar patterns in the NMI similarity comparison from extracted miRNA modules in Fig. 4. Furthermore, all subtypes dysregulation analyses showed high NMI similarity with the miRNA family assignments without having incorporated this prior knowledge. This implies that despite possible false-positives in identifying miRNA-target dysregulations, the pruned MDSN can still be an excellent tool to reveal miRNA-miRNA functional synergism when inferring novel miRNA functions.

## Conclusions

By utilizing a dysregulation metric that allows for analysis of multiple cancer subtypes, we proposed a pipeline to cluster miRNAs with high functional synergism. The extracted miRNA modules, when applied to grouped feature selection, can improve phenotype prediction and result in biomarkers with high precision and recall rate to known LUAD-associated miRNAs. Furthermore, the predicted miRNA modules extracted from different subtype analyses can be used to reveal common miRNA dysregulations across multiple subtypes in heterogeneous cancer types. Since miRNA-target dysregulations are implicated in many cancers, where multi-modal differential analyses between multiple cancer subtypes have mainly left undiscovered, we believe this tool can have broad applications in the development of new diagnosis and treatment strategies.

## Abbreviations

AUC: Area under the curve
Dys: Dysregulation
FDR: False discovery rate
LUAD: Lung adenocarcinoma
LUSC: Lung squamous cell carcinoma
MDSN: MicroRNA dysregulational synergistic network
miRNA: microRNA
mRNA: messengerRNA
NMI: Normalized mutual information
ROC: Receiver operating characteristic
RPKM: Reads per million mapped
SCC: Squamous cell carcinoma
SGL: Sparse group lasso
TCGA: The cancer genome atlas
WGCNA: Weighted gene co-expression network analysis
